# Standardising care in the ICU: a protocol for a scoping review of tools used to improve care delivery

**DOI:** 10.1186/s13643-020-01414-6

**Published:** 2020-07-19

**Authors:** Laura Allum, Chloe Apps, Nicholas Hart, Natalie Pattison, Bronwen Connolly, Louise Rose

**Affiliations:** 1 Florence Nightingale Faculty of Nursing, Midwifery and Palliative Care, King’s College London, London, UK; 2 Lane Fox Clinical Respiratory Physiology Research Centre, London, UK; 3 Physiotherapy department, St. Thomas’ Hospital, Guy’s and St. Thomas’ NHS Foundation Trust, Westminster Bridge Rd, London, UK; 4 Critical Care Research Group, St. Thomas’ Hospital, Guy’s and St. Thomas’ NHS Foundation Trust, Westminster Bridge Rd, London, UK; 5 National Institute for Health Research Biomedical Research Centre, Guy’s and St. Thomas’ NHS Foundation and King’s College London, London, UK; 6 University of Hertfordshire; East & North Herts NHS Trust; Florence Nightingale Foundation, London, UK; 7 Wellcome-Wolfson Institute for Experimental Medicine, Queen’s University Belfast, Belfast, UK; 8 Centre for Human and Applied Physiological Sciences, King’s College London, London, UK; 9 Department of Physiotherapy, The University of Melbourne, Melbourne, Australia

**Keywords:** Critical care, Patient care planning, Checklist, Delivery of Healthcare, Prolonged critical illness

## Abstract

**Background:**

Increasing numbers of critically ill patients experience a prolonged intensive care unit stay contributing to greater physical and psychological morbidity, strain on families and cost to health systems. Quality improvement tools such as checklists concisely articulate best practices with the aim of improving quality and safety; however, these tools have not been designed for the specific needs of patients with prolonged ICU stay.

The primary objective of this review will be to determine the characteristics including format and content of multicomponent tools designed to standardise or improve ICU care. Secondary objectives are to describe the outcomes reported in these tools, the type of patients and settings studied, and to understand how these tools were developed and implemented in clinical practice.

**Methods:**

We will search the Cochrane Library, the Cumulative Index to Nursing and Allied Health Literature (CINAHL), EMBASE, MEDLINE, PsycINFO, Web of Science, OpenGrey, NHS evidence and Trial Registries from January 2000 onwards. We will include primary research studies (e.g. experimental, quasi-experimental, observational and qualitative studies) recruiting more than 10 adult participants admitted to ICUs, high dependency units and weaning centres regardless of length of stay, describing quality improvement tools such as structured care plans or checklists designed to standardize more than one aspect of care delivery. We will extract data on study and patient characteristics, tool design and implementation strategies and measured outcomes. Two reviewers will independently screen citations for eligible studies and perform data extraction. Data will be synthesised with descriptive statistics; we will use a narrative synthesis to describe review findings.

**Discussion:**

The findings will be used to guide development of tools for use with prolonged ICU stay patients. Our group will use experience-based co-design methods to identify the most important actionable processes of care to include in quality improvement tools these patients. Such tools are needed to standardise practice and thereby improve quality of care. Illustrating the development and implementation methods used for such tools will help to guide translation of similar tools into ICU clinical practice and future research.

**Systematic review registration:**

This protocol is registered on the Open Science Framework, https://osf.io/, DOI 10.17605/OSF.IO/Z8MRE

## Background

Increasing numbers of patients remain in intensive care units (ICUs) for longer than a week due to increased survival rates, comorbidity and age in the general population resulting in lower resilience to acute illness and longer recovery [1–4]. Various terminology is used to describe these patients including ‘persistent critical illness’ and ‘chronic critical illness’ (CCI). Persistent critical illness is used to describe the point at which a patient’s presenting condition no longer predicts their risk of mortality [1]. Patients, however, continue to experience ongoing illness-related complications and organ failure [5]. This differentiates these patients from those experiencing prolonged respiratory weaning or conditions with an inherently long recovery time such as Guillain-Barré syndrome. Chronic critical illness is also used to describe patients with a prolonged length of stay (LOS) [2] and refers to a medically complex group of patients with multiple co-morbidities, often of older age. This frailty can result in less physical resilience to withstand the insult of critical illness [6]. For clarity, the term ‘prolonged ICU stay’ which encompasses both persistent and chronic critical illness, will be used in this paper to describe any patient with a stay of over 7 days. This timepoint is the lower threshold defined by Iwashyna et al. [7], who suggest that the transition to persistent critical illness occurs within a range of 7–22 days. These patients experience a range of complications, including muscle wasting [3] and long-term physical and functional deficits [8, 9], psychological distress [10], and cognitive deficits, leading to longer stays in hospital after ICU discharge [3] and prolonged recovery. They are more likely to die [11], and survivors are less likely to return home, often requiring ongoing nursing or residential care [12, 13]. Family members experience significant levels of psychological distress [14, 15], which may require involvement of social workers or psychologists.

The transition from acute critical illness to a prolonged ICU stay involves a shift in the goals of care. This frequently involves professions and specialities not typically involved in the acute phase of care, such as speech and language therapists, occupational therapists, social workers, and palliative care. The needs of this patient group are distinct and include rehabilitation. Family members require regularly updates and involvement in care [10]. Clinicians report feeling dissatisfied with their management of these patients due to the need to prioritise care for more unstable patients [12], a dislike of caring for lower acuity patients and a lack of training [16].

Intensive care units are complex environments, involving the coordination of multiple healthcare professions, specialties and numerous tasks for patients with life-threatening conditions [17]. Communication errors are common [18, 19] and contribute to patient harm and frustrations for clinicians [18, 20, 21]. In response to these errors, quality improvement tools including checklists, tools to structure ward rounds, bundles and protocols have been developed for the purposes of standardising care. These tools have the potential to improve safety [22, 23], patient, family and staff satisfaction [24], and understanding of goals of treatment [25–28], and can decrease ICU length of stay [17, 28]. However, these tools most commonly focus on medically orientated priorities of care delivery during the acute period of ICU admission [29–31]. Such tools may be less relevant to more stable patients requiring aspects of care delivery such as mobilisation, communication aids and patient-led goal setting [12, 32]. Additionally, many of these tools are designed for delivery of a single element of care, such as the prevention of infection associated with central line insertion, rather than the coordination of a range of tasks by the interprofessional team.

The ability of such tools to impact care is dependent on a wide range of factors [33], including successful implementation addressing local barriers to adoption, widespread ‘buy-in’ from clinicians and ongoing implementation strategies to sustain use [33]. A 2013 systematic review of the impact of knowledge translation studies in ICU concluded that there was insufficient evidence to identify the most effective knowledge translation strategies for improving practice in the ICU, particularly for quality improvement measures which cannot be protocolised—however, it did not include qualitative literature [34].

### Why is it important to do this review?

Given the demonstrable benefits of ICU quality improvement tools for patients in the acute phase of ICU admission, knowledge of the elements and factors that facilitate successful implementation could inform design of similar tools to address the distinct care needs of patients experiencing a prolonged ICU stay [32]. This is particularly important given the rising prevalence of these patients and their cost to the healthcare system. To the best of our knowledge, no review has synthesised this information.

Our primary objective is to determine the characteristics (i.e., format, content) of multicomponent tools designed to standardise and/or improve care delivery in adult ICU patient management. Our secondary objectives are to describe (1) what outcomes are reported, how they are measured and their effect; (2) the type of patients studied; (3) how tools were developed including patients and/or family member involvement; and (4) how these tools are implemented in practice.

We have chosen a scoping review approach as described by Tricco et al. [35] and adapted from Arksey and O’Malley [36] as the most appropriate methodology to achieve our objective of mapping the different components of these tools and the research on them to date. We also anticipate a range of different methodologies and study designs, for which scoping reviews are the advised methodology [36].

## Methods

The present protocol has been registered within the Open Science Framework platform (https://osf.io/z8mre). It is reported in accordance with the reporting guidance provided in the Preferred Reporting Items for Systematic Reviews and Meta-Analyses Protocols (PRISMA-P) statement [37]and the PRISMA extension for Scoping Reviews (PRISMA-ScR) [35] see PRISMA-P checklist in Additional file 1).

### Information sources and searches

A search strategy (Additional file 2) was created using a combination of MeSH terms and keyword combinations. We will adapt the search strategy for each database. We will search electronic databases including MEDLINE (Ovid), the Cumulative Index to Nursing and Allied Health Literature (CINAHL), EMBASE, PsycINFO and Web of Science from January 2000 onwards, to reflect current ICU care. We will search for ongoing or completed trials using the World Health Organisation International Clinical Trials Registry Platform (http://apps.who.int/trialsearch/) and systematic reviews via the Cochrane Library. We will search for grey literature using Opengrey (http://www.opengrey.eu/), NHS evidence (https://www.evidence.nhs.uk/), Google Scholar and Prospero. We will scan reference lists of included studies for other studies of relevance. We will exclude editorials, commentaries and animal studies. LA will contact authors where necessary for full-text papers or to enquire about unpublished work identified through protocols or trial registries. A draft search for MEDLINE (Ovid) is available in Additional file 2.

### Eligibility criteria

Articles will be selected according to the following criteria for study population, intervention and comparator and study design.

### Population

We will include studies recruiting adult patients aged 18 years and older admitted to an intensive or critical care unit, high dependency or a weaning centre, respiratory care unit or long-term acute care hospital (LTACH), regardless of length of stay. We will also include studies that include family members/caregivers and the healthcare practitioners responsible for the care of these patients as participants.

### Intervention

We will include studies that report on quality improvement interventions such as multicomponent structured care plans, goal sheets, or checklists designed to standardize or remind clinicians about more than one aspect of care delivery. We will exclude checklists for procedures such as central line insertion or tools such as care bundles with single objectives of care, e.g. to prevent ventilator-associated pneumonia. Protocols, including those for delirium prevention, sedation management, weaning, and mobilisation will also be excluded as we do not seek to produce a decision algorithm and therefore this format is outside the scope of this review. Examples of such tools are daily goals sheets [38, 17] and rounding checklists [31].We will exclude: 1. checklists for procedures such as central line insertion; 2. care bundles with single objectives of care e.g. to prevent ventilator-associated pneumonia; 3. Single objective protocols, including those for delirium prevention, sedation management, weaning, and mobilisation will also be excluded as we do not seek to produce a decision algorithm and therefore this format is outside the scope of this review. 

### Comparators

We will include studies with an active comparator (i.e. another quality improvement tool), a passive comparator (i.e. usual care) and no comparator.

### Outcomes

We will not make decisions related to inclusion of studies based on outcomes reported.

### Study design

We will include all qualitative and quantitative study designs including experimental, quasi-experimental, observational studies and qualitative studies except case series, as this design is not appropriate for the evaluation of a Quality Improvement (QI) tool. For pragmatic reasons, we will only include studies published in English. We will include unpublished work in the form of conference proceedings and theses.

### Study selection

One author (LA) will independently screen titles and abstracts to remove obvious exclusions using Endnote X8. The full text of citations selected for potential inclusion will be retrieved and assessed independently by two authors (LA and CA) for eligibility, with a third reviewer (LR) available for arbitration if needed. All decisions will be recorded in an Excel file.

### Data charting process

Two authors (LA and CA) will independently extract data using a Google form (Additional File 3) iteratively designed for this review by three authors (LA, CA and LR). The form captures the following variables:

- Participants—participant characteristics (patient/family member/healthcare worker), country, setting (e.g. ICU or weaning unit), admitting speciality).
- Study design—study type and methodology used
- Tool—the type, design, frequency of completion, content and aims of the QI tool described
- Tool development—we will extract data on how the tool was developed, including patient and family involvement and feasibility testing,
- Tool implementation—methods used and evaluated using the Theoretical Domains Framework [39] to describe barriers and facilitators to adoption.
- Outcomes—the effect of the interventions will be extracted including descriptive data and outcome measures used.

Differences in extracted data between the two reviewers will be resolved by discussion, and a third reviewer (LR) consulted if an agreement cannot be reached. LA will contact corresponding authors for missing information as needed (e.g. tool development or implementation strategies).

### Critical appraisal of evidence sources

We will use the results of the MMAT [40] to help inform conclusions and recommendations from the scoping review, as a robust tool for describing quality of mixed-methods studies. The authors of the tool strongly discourage using stand-alone numerical scores for studies, and so we intend to use the tool as they advise to describe high-, medium- and low-quality studies [41].

### Evidence synthesis, analysis and interpretation

We will complete a PRISMA study flow diagram [37] to describe our search results. We will provide a narrative synthesis to describe our findings as recommended by Levac et al. [42].

We will summarise characteristics of included studies, including the setting (type of unit and country) and type of tool used using descriptive statistics.

To address our primary objective, we will present tables grouped according to tool type (e.g. checklist, care plan, goal sheet) describing the purpose and content of each tool. We will include reported outcomes, their measures (if applicable) and the effects of the intervention on these outcomes.

To address our secondary objectives, a table will be produced describing the patients and settings studied in each included tool, and tool development including source of content (e.g. published evidence, expert opinion) and whether patients and family members were involved in its development. Data on methods of tool implementation will be summarised with reported barriers and facilitators interpreted using the Theoretical Domains Framework.

## Discussion

International work [32] to identify and prioritise the processes of care that most improve the experience of patients with prolonged ICU stay and their families is ongoing, using principles of experience-based co-design. The findings of this review will inform the design and implementation of quality improvement tools to improve/standardise the delivery of these identified processes of care, to assist knowledge translation to clinical practice.

A previous systematic review [32] failed to identify any quality improvement tools specific to long stay patients in intensive care. An improved comprehension of how such tools are developed and implemented will help to guide translation for similar tools in ICU.

Limitations of our findings are anticipated due to heterogeneity in the tools studied and differences in the context in which they are applied, and the selection bias inherent with only including papers published in English, and after 2000. Limitations of scoping review methodology will mean that a synthesis of findings across articles is not possible.

This review will also provide a summary for clinicians seeking to better understand and utilise the range of quality improvement tools used to improve more than one aspect of care in the ICU.

## Supplementary information

**Additional file 1.** PRISMA extension for scoping reviews checklist (PRISMA-scr).

**Additional file 2.** MEDLINE search strategy.

**Additional file 3.** Date extraction document.

## Data Availability

Not applicable.

## Abbreviations

ICU: Intensive care unit
CCI: Chronic critical illness
LOS: Length of stay
PRISMA-scr: Preferred Reporting Items for Systematic Reviews and Meta-Analyses, Scoping Review
MeSH: Medical Subject Headings
LTACH: Long-term acute care hospital
QI: Quality improvement
