# Supplementary material for: Standardising care in the ICU: a protocol for a scoping review of tools used to improve care delivery
Source: Syst Rev. 2020 Jul 19;9:164. doi: 10.1186/s13643-020-01414-6 (PMC7368855; doi:10.1186/s13643-020-01414-6)
Supplement: Supplementary file 2 — Additional file 2. MEDLINE search strategy. [file 13643_2020_1414_MOESM2_ESM.docx]

critDatabase: Ovid MEDLINE(R) and Epub Ahead of Print, In-Process & Other Non-Indexed Citations and Daily

Search Strategy:

--------------------------------------------------------------------------------

1 Critical Care/

2 Intensive Care Units/ or burn units/ or coronary care units/ or respiratory care units/

3 Critical Illness/

4 ((intensive or critical or acute) adj3 care).tw,kf.

5 (ICU or ICUs or SICU or SICUs or CCU or CCUs).tw,kf.

6 (burn* adj3 (unit? or centr* or center*)).tw,kf.

7 ((cardiac or coronary or heart) adj3 (unit? or centr* or center*)).tw,kf.

8 (respiratory adj3 (unit? or centr* or center*)).tw,kf.

9 (critical* adj ill*).tw,kf.

10 (high dependency adj3 (unit? or centr* or center*)).tw,kf.

11 ((stepdown or step-down) adj3 (unit? or centr* or center*)).tw,kf.

12 (HDU or SDU or EDSDU or HDUs or SDUs or EDSDUs).tw,kf.

13 (speciali?ed weaning adj3 (unit* or centr* or center*)).tw,kf.

14 1 or 2 or 3 or 4 or 5 or 6 or 7 or 8 or 9 or 10 or 11 or 12 or 13

15 Checklist/

16 Goals/

17 Patient Care Planning/

18 Patient Care Bundles/

19 Needs Assessment/

20 Continuity of Patient Care/

21 Interdisciplinary Communication/

22 Decision Support Systems, Clinical/

23 Decision Support Techniques/

24 Practice guideline/

25 Daily goals.tw,kf.

26 (Goal? adj3 (set* or plan*)).tw,kf.

27 (decision adj3 (aid or aids or app or apps or application)).tw,kf.

28 (decision adj3 (tool or tools)).tw,kf.

29 ((electronic or physical) adj3 tool?).tw,kf.

30 (quality adj3 bundle?).tw,kf.

31 ((electronic or physical) adj3 tool?).tw,kf.

32 problem list?.tw,kf.

33 Plan? of care.tw,kf.

34 (Care adj3 plan?).tw,kf.

35 ((patient* or interdisciplinary) adj3 round?).tw,kf.

36 (Multidisciplinary adj3 round?).tw,kf.

37 (Patient? adj3 Goal?).tw,kf.

38 (Decision adj3 (support or framework)).tw,kf.

39 shared decision making.tw,kf.

40 (outcome and process assessment).tw,kf.

41 Process of care.tw,kf.

42 Quality improvement/

43 Quality indicators, Health Care/

44 quality assurance, health care/ or benchmarking/ or practice guidelines as topic/

45 "quality of health care"/ or "process assessment (health care)"/

46 Quality metrics.tw,kf.

47 Quality indicators.tw,kf.

48 (quality adj3 (assurance or improvement or management)).tw,kf.

49 15 or 16 or 17 or 18 or 19 or 20 or 21 or 22 or 23 or 24 or 25 or 26 or 27 or 28 or 29 or 30 or 31 or 32 or 33 or 34 or 35 or 36 or 37 or 38 or 39 or 40 or 41

50 42 or 43 or 44 or 45 or 46 or 47 or 48

51 49 or 50

52 14 and 51

53 limit 52 to (english language and yr="1999 -Current")

54 exp Child/ not exp Adult/

55 exp Infant/ not exp Adult/

56 exp adolescent/ not exp Adult/

57 53 not (54 or 55 or 56)

58 exp Animals/ not Humans/

59 57 not 58

60 (comment or editorial or news or newspaper article or letter).pt.

61 59 not 60
